# Valgus Tibial Component Placement: A Novel Risk Factor for Tibial Fracture in Cementless Oxford® Partial Knee Arthroplasty

**DOI:** 10.7759/cureus.111330

**Published:** 2026-06-22

**Authors:** Tummala Mohit, Abdulaziz A Basaqr, Takafumi Hiranaka, Takaaki Fujishiro

**Affiliations:** 1 Department of Orthopaedic Surgery and Joint Surgery Centre, Takatsuki General Hospital, Takatsuki, JPN; 2 Department of Orthopaedic Surgery, Krishna Institute of Medical Sciences, Kondapur, Hyderabad, IND

**Keywords:** cementless oxford partial knee, medial unicompartmental knee arthroplasty, periprosthetic tibial fracture, valgus subsidence, valgus tibial component

## Abstract

The Oxford® Partial Knee (OPK; Zimmer Biomet, Warsaw, IN, USA) is a widely used unicompartmental knee arthroplasty (UKA) system. We report a case in which valgus malpositioning of the tibial component relative to the resection surface may have contributed to an early postoperative fracture.

A 70-year-old woman underwent bilateral cementless OPK arthroplasty. On the left side, the tibial component was inserted in valgus alignment relative to a slight varus resection. Postoperative CT revealed a non-displaced tibial fracture. Internal fixation was performed, and bone healing proceeded uneventfully. The fracture was likely caused by valgus alignment of the component relative to the tibial cut, producing an eccentric loading, thereby generating a wedge effect and rotational force. Contributing factors for this malpositioned component placement may have included inadequate osteophyte removal, oversized component impingement on the medial collateral ligament (MCL), meniscal cuff interposition, and malaligned keel slot preparation. Bone union was achieved with stable fixation and biological bone ingrowth.

Valgus malpositioning of the tibial component relative to the resection surface represents a previously unreported risk factor for periprosthetic tibial fracture in cementless OPK. Careful intraoperative technique can reduce this risk, and bone healing remains achievable following fracture.

## Introduction

The cementless Oxford® Partial Knee (OPK; Zimmer Biomet, Warsaw, IN, USA) was introduced nearly two decades ago. This implant offers several notable advantages, including the elimination of cement-related complications such as excessive cement application, suboptimal cement mantling, loose cement fragments, and third-body wear. Additionally, the cementless OPK demonstrates fewer radiolucent lines, shorter operative times, and improved implant longevity and clinical outcomes [1-3].

Nevertheless, the cementless Oxford® unicompartmental knee arthroplasty (UKA) is associated with complications such as valgus subsidence and periprosthetic fractures. Valgus subsidence is reported in 1.6%-8.2% of cases [4-6], usually stabilizing within six months without intervention and with clinical outcomes remaining favorable. In contrast, periprosthetic fractures are more serious, often requiring revision or fixation. Reported fracture incidence ranges from 1.2%-1.6% [3,7,8] in Western populations, but significantly higher rates of 3.8%-8.0% [9-11] have been observed in Asian populations.

These fractures have been attributed to a combination of implant-, surgical-, and patient-related factors. An implant-related factor includes cementless components with excessive interference fit [1,12]. Patient-related factors include female gender, osteoporosis, small tibial size, medial tibial condylar overhang [10, 13], and excessive proximal tibial vara [14-16]. Surgical factors like forceful impaction or a heavy hammer, and pin-related factors like a medially placed pin, excessive pinholes, or no pre-drilling. Errors in tibial preparation, such as recuts in the tibia, uneven cuts, excessively medial low tibial cuts, and deep vertical/posterior cuts, contribute to the fracture risk. Reduced keel-to-cortex distance [14] and inadequate lateralization of the tibial component close to the tibial spine have all been implicated as contributory factors. We present a case from our institution that suggests valgus malpositioning of the tibial component, an unreported factor, as a potential novel risk factor for periprosthetic tibial fracture. 

## Case presentation

A 70-year-old woman presented with bilateral chronic medial knee pain, more severe on the left, persisting for over a decade. Conservative management had failed, significantly impacting daily activities. Radiographs (Figure 1a) confirmed bilateral anteromedial osteoarthritis. MRI (Figure 1b) was used to assess the lateral compartments and the anterior cruciate ligament (ACL) integrity. Preoperative left knee scores were the following: range of motion (ROM) 0°-150°, Oxford Knee Score 32, Knee Society Score (KSS) 70, and Function Score 35.

**Figure 1:**
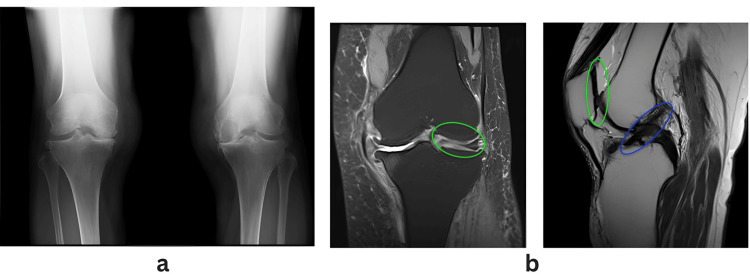
Preoperative imaging a: Preoperative anteroposterior weight-bearing radiograph showing medial compartment osteoarthritis. b: MRI of the left knee showing the status of the lateral and patellofemoral compartment cartilage (green encircled) and intact anterior cruciate ligament bundles (blue encircled).

Following counselling, she underwent single-stage bilateral cementless OPK arthroplasty. The procedure was generally performed according to the surgical technique manual provided by the manufacturer. For the tibial bone cut, however, a custom varus tibial cutting jig was used, as previously reported, with a target inclination of approximately 3° of varus. No intraoperative fracture or obvious technical difficulty was recognized during the procedure. A size 3 tibial component was implanted on the left knee (size 4 on the right knee).

Postoperative radiograph (Figure 2) revealed a medial overhang of the portion of the tibial component and showed a 5° valgus alignment relative to the 2° varus resection surface (Figure 3). Sagittal alignment was acceptable. She was mobilized on the same day with full weight-bearing and unrestricted ROM.

**Figure 2:**
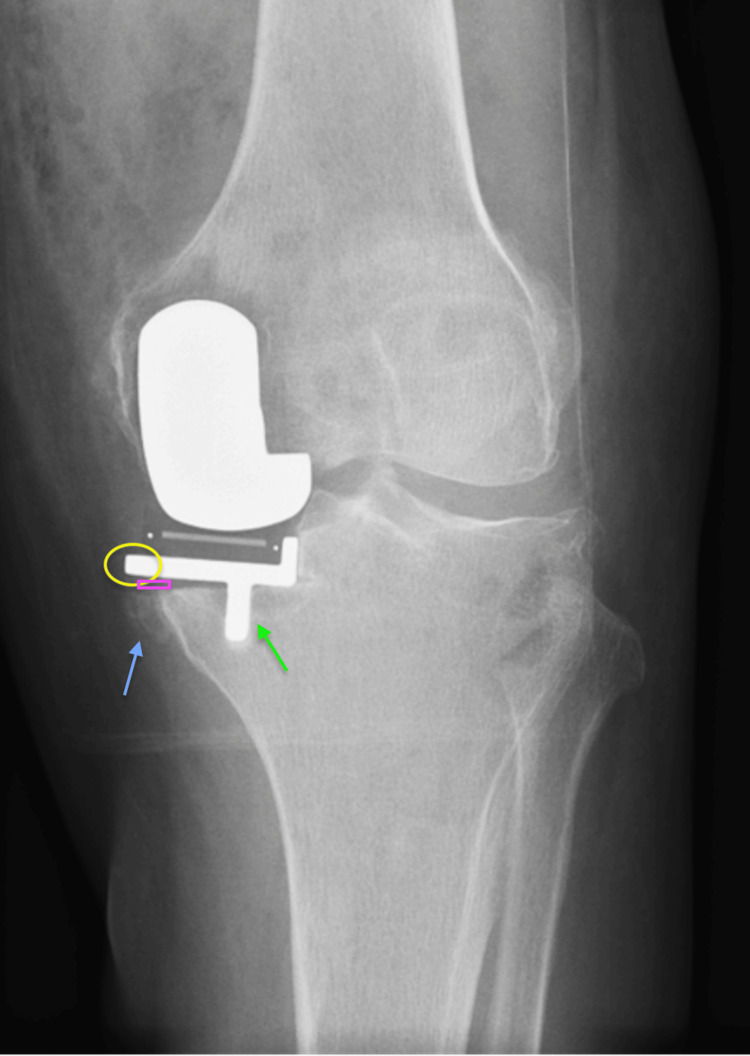
Postoperative Imaging Immediate postoperative radiographs of the left knee shows medial overhang of the tibial component (yellow encircled) with the medial edge resting on residual medial tibial osteophytes (blue). The tibial component aligned in valgus against the tibial cutting plane; this could have been due to interposed remnants of the medial meniscus (pink) or an external rotational error during keel slot preparation (green).

**Figure 3:**
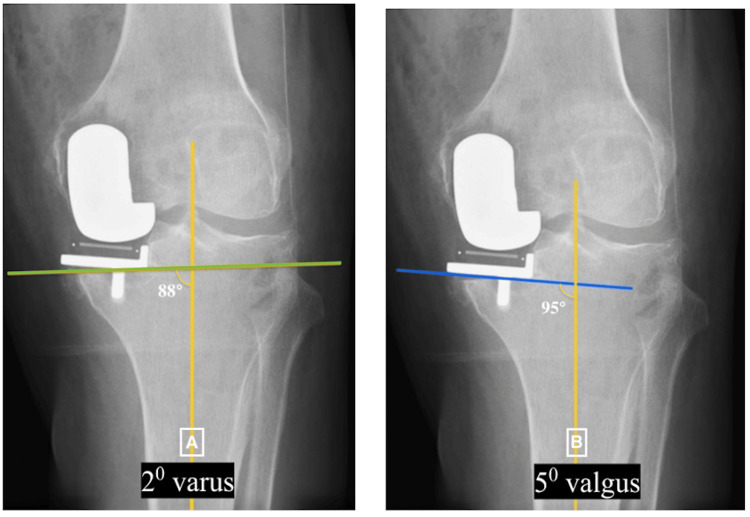
Valgus malposition of the tibial component A: Radiographs demonstrate a tibial cut oriented at 2° of varus relative to the tibial axis, while B: the immediate postoperative X-ray shows the tibial component positioned at 5° of valgus orientation.

Within days, she developed disproportionate pain in the left knee. CT imaging (Figure 4) revealed non-displaced fracture lines extending from the posterior keel to the medial and posteromedial cortices. Open reduction and internal fixation were performed using a TomoFix plate (DePuy Synthes, Oberdorf, Switzerland). Because the plate was inserted through an extension of the original UKA incision, it was positioned somewhat anteromedially. Although the plate was not centered over the posteromedial fracture fragment, it remained in contact with the anterior portion of the fragment in an eccentric position. This eccentric contact, combined with the fixed-angle locking construct, provided buttress support against shear forces and valgus displacement. Fracture union was subsequently achieved without loss of fixation. (Figure 5). The medial collateral ligament (MCL) was preserved, and full weight-bearing was resumed immediately post fixation.

**Figure 4:**
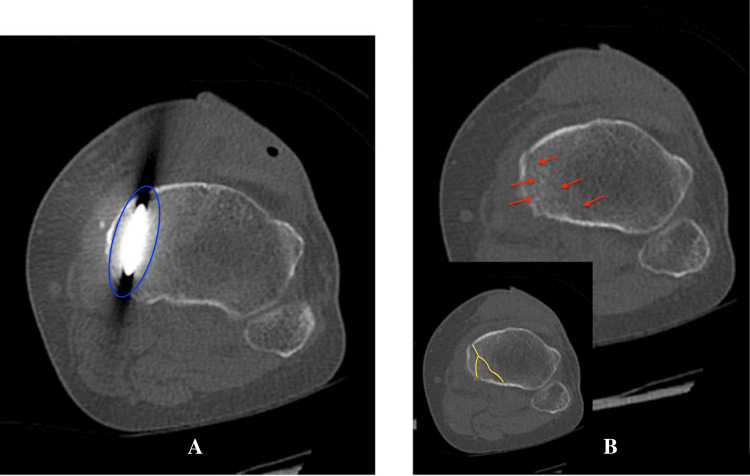
Detection of the tibial fracture on CT scan Axial view of the CT scan shows (A) the location of the tibial component keel (blue) and (B) the medial condyle fracture lines (red arrows and yellow demarcation in the insert image).

**Figure 5:**
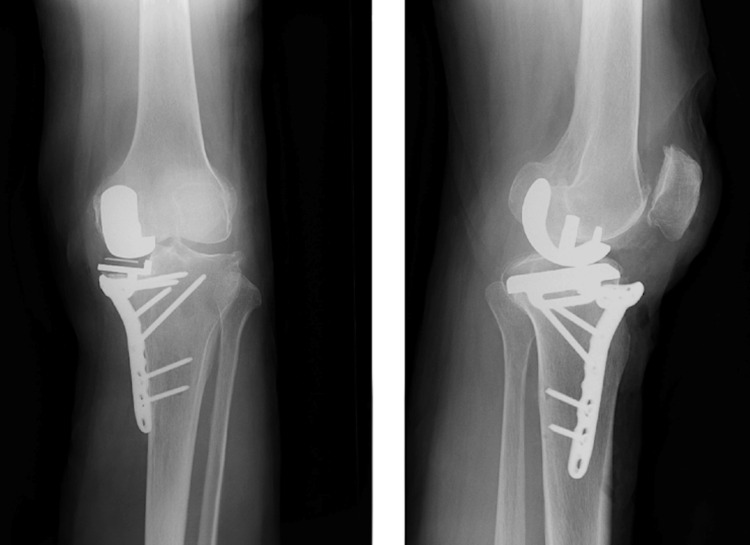
Post-fixation imaging Anteroposterior and lateral images of the left knee after open reduction and plate fixation with a 3.5mm Tomofix plate.

Recovery was uneventful. Notably, the tibial component exhibited progressive valgus subsidence starting a week before fixation and stabilizing by nine months, eventually conforming to the resection surface (Figure 6). At two-year follow-up, the patient had a left knee ROM of 0°-155°, Oxford Score of 41, KSS of 95, Function Score of 80, and a Forgotten Joint Score of 100. Radiographs confirmed complete union, absence of lucency at the bone-implant interface, and complete seating of the component with a minor increase in valgus alignment (Figure 7).

**Figure 6:**
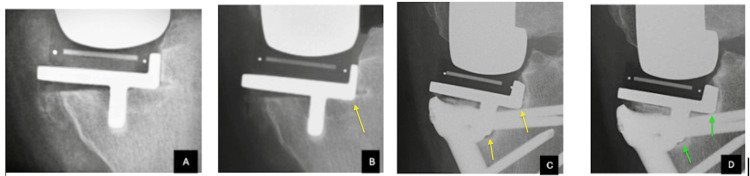
Valgus subsidence of the tibial component Radiographs of the left knee: (A) Immediate postoperative radiograph; (B) One week postoperatively, prior to plate fixation, showing minimal subsidence without any radiographically visible fracture (yellow arrow); (C) Three month follow-up demonstrating progressive valgus subsidence (yellow arrows); (D) Nine month follow-up showing stabilized alignment with the tibial component well-seated and conforming to the tibial resection plane (green arrows).

**Figure 7:**
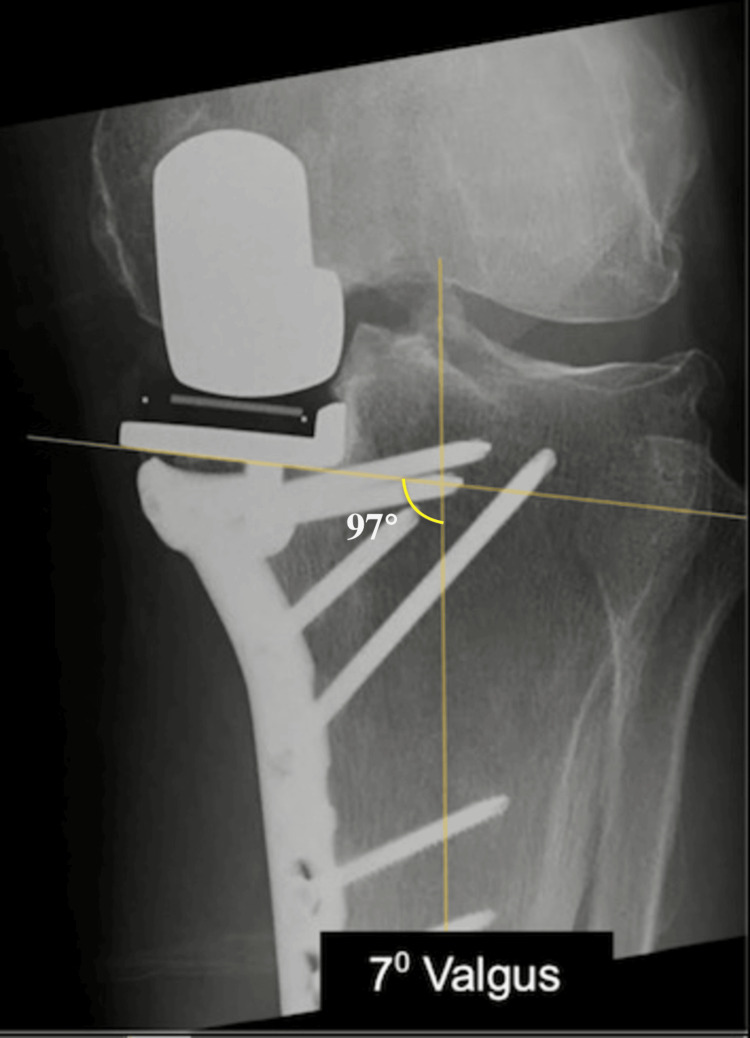
Final follow-up imaging Final follow-up radiograph shows a valgus subsidence with the tibial component aligned at 7 degrees of valgus to the tibial axis.

## Discussion

We report a rare yet suggestive case of early periprosthetic tibial fracture following valgus malpositioning of the tibial component relative to the resection surface in a cementless OPK. To our knowledge, this is the first report to specifically implicate valgus implant alignment relative to the tibial cut surface as a novel risk factor for postoperative fracture.

The orientation of the tibial cut is an important factor influencing fracture risk after Oxford® UKA. A valgus tibial cut has been reported to increase stress on the medial cortex and reduce the keel-to-cortex distance, thereby increasing the risk of tibial fracture [14,17]. Conversely, a varus tibial cut may reduce medial cortical stress by increasing cortical support around the keel. In the present case, the tibial cut was made in slight varus; therefore, the cut orientation itself was unlikely to be the primary cause of the fracture. However, the keel-to-cortex distance was only 2.9 mm, indicating that the tibial keel was located close to the medial tibial cortex. This short distance may have increased stress concentration at the medial cortex, particularly when combined with valgus placement of the cementless tibial component relative to the prepared tibial bone surface. 

Although no obvious technical difficulty was recognized intraoperatively, retrospective review of the postoperative images suggested that the tibial component was malpositioned relative to the prepared tibial bone surface. Possible technical mechanisms include rotational error during keel-slot preparation, eccentric impaction, and incomplete or asymmetric seating of the component. However, because these factors were identified retrospectively, the precise intraoperative mechanism responsible for the final component position could not be determined.

Instead, the implant was placed in valgus relative to the cut surface. This mismatch created an eccentrically loaded interference fit, generating a wedge-like effect. The lateral corner of the component exerted a separating force between the central keel and the medial tibial condyle. Additionally, rotational force from the unsupported medial aspect, which later settled onto the tibial cut, may have further contributed to fracture propagation (Figure 8). In contrast, varus implantation would likely create compressive forces at the bone-implant interface. Any minor gaps between the cementless component and bone could then be filled in with new bone, eventually achieving biological bonding (Figure 9). Based on this biomechanical rationale, valgus malpositioning of the implant was deemed the primary cause of the fracture. It should be emphasized that the mechanism of fracture in this case was likely multifactorial. Although valgus placement of the cementless tibial component relative to the prepared tibial bone surface may have contributed to localized stress concentration at the medial tibial cortex, other factors may also have played a role, including the short keel-to-cortex distance, possible rotational or translational component malposition, and limitations related to radiographic projection. Therefore, valgus component placement should be interpreted as a possible contributing factor rather than as a proven independent cause of fracture.

**Figure 8:**
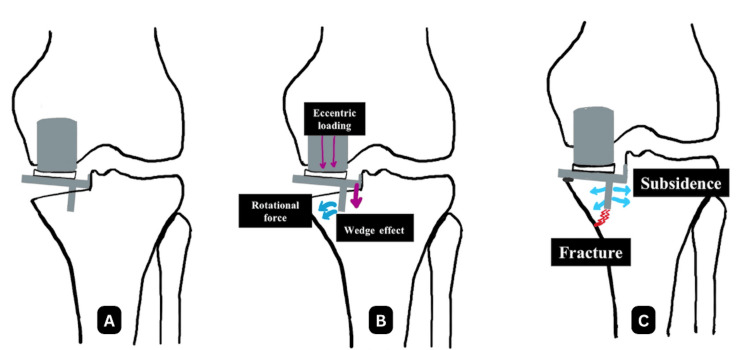
Schematic illustration of the proposed mechanism leading to the tibia fracture (A) Valgus malpositioning of the tibial component. (B) Eccentrically loaded interference fit of the cementless component (purple arrows), the mismatch between the cut and the component placement created a wedge-like effect (bold purple arrow) where the lateral corner of the implant exerted a separating force between the central body and the medial condyle of the tibia. Additionally, there was a rotational force (blue arrows) from the unsupported medial portion of the implant. This axial loading across the eccentrically placed component generates asymmetric stress, potentially converting compressive forces into distractive forces along the medial tibial condyle. (C) This could have resulted in a medial condyle fracture (red marked), accompanied by valgus subsidence (blue arrows) of the tibial component. Image credit: Dr. Tummala Mohit; Manually illustrated using Microsoft PowerPoint (Microsoft Corp., Redmond, WA, USA) and reproduced with permission from the author.

**Figure 9:**
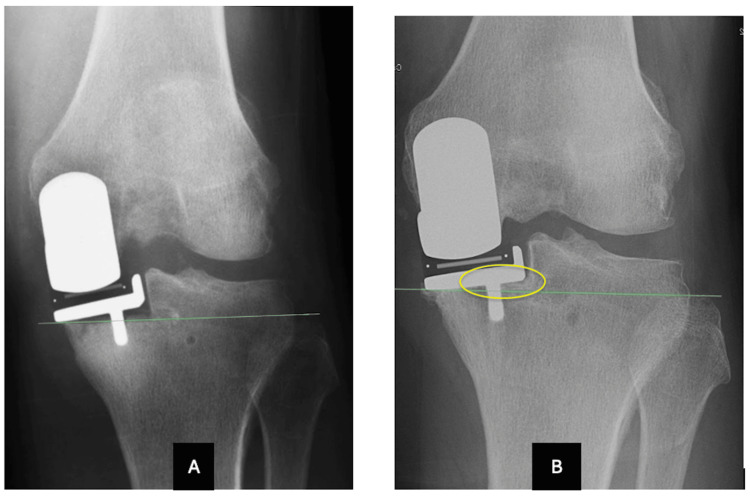
Varus positioned tibia component (another patient) Immediate postoperative radiograph (A) showing a varus-positioned tibial component in another patient, with no evidence of fracture. Follow-up radiograph at two years (B) demonstrates stable implant positioning with biological bonding and good osteointegration under the cementless prosthesis. Note: Published with the patient's written informed consent.

Several intraoperative factors may have led to this malalignment, as shown in Figure 2. First, residual medial tibial osteophytes, left to preserve the MCL, can prevent full seating and mimic the tibial margin. Second, oversized components may cause medial overhang and MCL impingement, pushing the implant into valgus. Third, interposed remnants of the medial meniscus may disrupt full seating. Fourth, external rotational error during keel slot preparation may guide the implant into valgus. 

With regards to the management [18], we opted for internal fixation. While conservative treatment avoids surgery, it requires prolonged non-weight-bearing and carries a higher risk of displacement. Internal fixation allows early mobilization and preserves the existing implant. Total knee arthroplasty (TKA), although definitive, is more invasive and typically requires stemmed components. Our institutional approach is to prefer conservative treatment for nondisplaced fractures confirmed on CT/MRI and to consider internal fixation when adequate cortical support and fragment size are present. TKA is reserved for cases prioritizing early full weight-bearing after initial treatment failure [19] or minimizing long-term risk [20].

Interestingly, this case also showed valgus subsidence of the tibial component. Beginning one week before fixation and stabilizing over nine months, the implant gradually conformed to the resection surface as serially depicted in Figure 6. Final follow-up, as can be seen in Figure 7, showed 7° valgus alignment. Valgus subsidence aided tibial component seating and conformity. The fracture healed without displacement, with good bone ingrowth, demonstrating achievable implant bonding post fracture, a potential advantage of cementless OPK.

This case report has several limitations. First, because this was a single-case report, the independent causal role of valgus tibial component placement could not be definitively established. Second, pre- and postoperative limb alignment could not be directly compared under identical radiographic conditions, and the interpretation of component position may have been influenced by radiographic projection and limb rotation. Third, rotational or translational malposition of the tibial component may also have contributed to the fracture mechanism. Therefore, the fracture should be interpreted as a multifactorial event, with valgus placement of the cementless tibial component considered as one possible contributing factor.

## Conclusions

Valgus placement of the cementless tibial component relative to the prepared tibial resection surface may represent a previously unreported contributing factor for early periprosthetic tibial fracture after Oxford® UKA. This malposition likely produces an eccentrically loaded interference fit, generating a wedge-like effect and rotational forces along the medial tibial cortex. Nevertheless, the fracture should be regarded as a multifactorial event, and careful intraoperative technique, including thorough osteophyte removal, appropriate component sizing, clearance of meniscal remnants, and accurate keel-slot preparation, may reduce this risk. When a fracture does occur, internal fixation allows early mobilization while preserving the existing implant, and bone healing with stable osseointegration of the cementless component remains achievable.
